# Enhanced osteochondral repair by leukocyte-depleted platelet-rich plasma in combination with adipose-derived mesenchymal stromal cells encapsulated in a three-dimensional photocrosslinked injectable hydrogel in a rabbit model

**DOI:** 10.1186/s13287-024-03750-z

**Published:** 2024-06-03

**Authors:** Tomoya Iseki, Benjamin B. Rothrauff, Shinsuke Kihara, Kalon J. Overholt, Tarek Taha, Hang Lin, Peter G. Alexander, Rocky S. Tuan

**Affiliations:** 1 Center for Cellular and Molecular Engineering, Department of Orthopaedic Surgery, University of Pittsburgh School of Medicine, Pittsburgh, PA 15219 USA; 2 Department of Orthopaedic Surgery, Hyogo Medical University, 1-1 Mukogawa-cho, 663-8501 Nishinomiya City, Hyogo Japan; 3 Institute for Tissue Engineering and Regenerative Medicine, The Chinese University of Hong Kong, Hong Kong, SAR China

**Keywords:** Mesenchymal stem/stromal cells, Platelet-rich plasma, Cartilage regeneration

## Abstract

**Introduction:**

Intra-articular injection of adipose-derived mesenchymal stromal cells (ASCs) and/or platelet-rich plasma (PRP) have been reported to independently and synergistically improve healing of osteochondral lesions in animal models. However, their independent and combined effects when localized to an osteochondral lesion by encapsulation within a photocrosslinkable methacrylated gelatin hydrogel (GelMA) have not been explored. Herein we investigated a unique combination of allogeneic ASCs and PRP embedded in GelMA as a single-stage treatment for osteochondral regeneration in a rabbit model.

**Methods:**

Thirty mature rabbits were divided into six experimental groups: (1) Sham; (2) Defect; (3) GelMA; (4) GelMA + ASCs; (5) GelMA + PRP; and (6) GelMA + ASCs + PRP.At 12 weeks following surgical repair, osteochondral regeneration was assessed on the basis of gross appearance, biomechanical properties, histological and immunohistochemical characteristics, and subchondral bone volume.

**Results:**

In terms of mechanical property reflecting the ability of neotissue to bear stress, PRP only group were significantly lower than the Sham group (*p* = 0.0098). On the other hand, ASCs only and ASCs combined with PRP groups did not exhibit significantly difference, which suggesting that incorporation of ASCs assists in restoring the ability of the neotissue to bear stresses similarly to native tissue (*p* = 0.346, *p* = 0.40, respectively). Safranin O in ASCs combined with PRP group was significantly higher than the Defect and GelMA only groups (*p* = 0.0009, *p* = 0.0017, respectively). Additionally, ASCs only and ASCs combined with PRP groups presented especially strong staining for collagen type II. Surprisingly, PRP only and PRP + ASCs groups tended to exhibit higher collagen type I and collagen type X staining compared to ASCs only group, suggesting a potential PRP-mediated hypertrophic effect.

**Conclusion:**

Regeneration of a focal osteochondral defect in a rabbit model was improved by a single-stage treatment of a photocrosslinked hydrogel containing allogenic ASCs and autologous PRP, with the combination of ASCs and PRP producing superior benefit than either alone. No experimental construct fully restored all properties of the native, healthy osteochondral unit, which may require longer follow-up or further modification of PRP and/or ASCs characteristics.

**Supplementary Information:**

The online version contains supplementary material available at 10.1186/s13287-024-03750-z.

## Introduction

Trauma-induced osteochondral injury is a predisposing factor for osteoarthritis (OA) due to the poor innate healing capacity of cartilage [1, 2]. Although several clinical procedures for treating focal cartilage defects exist, including microfracture, osteochondral autograft or allograft transplantation, and autologous chondrocyte implantation (ACI), the effectiveness of these procedures to restore articular hyaline cartilage remain respectively limited by long-term phenotypic changes, inadequate graft availability and associated donor site morbidity, and limited cell numbers with chondrogenic dedifferention during ex vivo expansion [3]. Therefore, tissue engineering approaches, including the independent or combined use of cells, scaffolds, and growth factors to more consistently restore the native structure and function of osteochondral defects, are currently being explored in an effort to improve upon presently available clinical treatments.

Hydrogels are a class of bioscaffold that can form 3-dimensional networks that possess clinically desirable properties of biocompatibility, bioadhesiveness, and biodegradability [4–6]. Additionally, hydrogels can be delivered as a liquid solution to fill irregularly shaped defects [7, 8]. Recent studies have shown that injectable hydrogels can support intraoperative cell seeding, allowing for point-of-care fabrication of a new matrix that approaches the biomechanical properties of the surrounding cartilage [9]. An injectable methacrylated gelatin hydrogel (GelMA) scaffold is capable of undergoing polymerization upon exposure to ultraviolet (UV) light, and these scaffolds have been shown to support chondrogenic differentiation of embedded cells [10, 11]. Therefore, in situ photopolymerization of cell-seeded GelMA scaffolds could be utilized during arthroscopic surgical procedures, offering a minimally invasive alternative to current cartilage repair techniques employing large arthrotomies and open dissection.

Platelet-rich plasma (PRP) is a blood derivative, largely devoid of red blood cells, which has been increasingly investigated as an autologous source of growth factors to promote tissue healing, including cartilage defects and bone defects [12–14]. Notably, PRP contains growth factors such as transforming growth factor-β (TGF-β), vascular endothelial growth factor (VEGF), platelet-derived growth factor (PDGF), insulin growth factor-1 (IGF), and basic fibroblastic growth factor (b-FGF), all of which have been shown to affect tissue regeneration. More specifically, PRP has been shown to promote in vitro chondrogenic differentiation, although this effect is variable and likely dependent upon PRP composition [14–17]. Additional studies have demonstrated that PRP can be synergistically combined with scaffolds to control growth factor release and promote reparative cell recruitment, in turn improving tissue repair [18, 19].

Mesenchymal stromal cells (MSCs) are an alternative cell source to chondrocytes, as currently used clinically in ACI. In comparison to chondrocytes, MSCs offer a higher ease of isolation, higher proliferation rate, and can undergo both chondrogenic and osteogenic differentiation [20, 21]. Adipose-derived mesenchymal stromal cells (ASCs) are found in higher concentrations than bone marrow-derived mesenchymal stromal cells (BMSCs) as a proportion of the heterogeneous cell population derived from lipoaspirate or bone marrow aspirate, respectively, therefore representing a promising source of MSCs. ASCs may improve osteochondral repair by differentiating into chondrocytes and/or bone with associated neotissue formation but, more likely, induce regeneration through paracrine mediators [22, 23]. Intra-articular injection of ASCs and/or PRP have been reported to independently and synergistically improve healing of osteochondral lesions in animal models [24–26] but their independent and combined effects when localized to an osteochondral lesion by encapsulation within a photocrosslinkable GelMA hydrogel have not been explored.

The purpose of this study was to test whether a combination of ASCs and PRP encapsulated within a 3-dimensional photocrosslinked injectable methacrylated gelatin hydrogel is beneficial for focal osteochondral defect repair in a rabbit model. We hypothesized that hydrogel supplementation with PRP or ASCs would enhance osteochondral repair, with the combination of PRP and ASCs yielding synergistic effect for superior healing.

## Methods and materials

### Cell characterization and isolation

Adipose-derived stromal cells (ASCs) were obtained from rabbit subcutaneous adipose tissue of the interscapular region in six post-breeder New Zealand white rabbits (6-month-old female, averaging 4.0 kg bodyweight, with an average of 30 g adipose tissue per rabbit) under an IACUC-approved and –exempted protocol (University of Pittsburgh). The adipose tissue was digested with 2 mg/mL type I collagenase (Worthington Biochemical, Lakewood, NJ), 1 mg/mL trypsin (Invitrogen, Carlsbad, CA), and 2% antibiotic-antimycotic (Invitrogen) in Dulbecco’s Modified Eagle’s Medium (DMEM; Invitrogen) at 37 °C for 3 h with 1 g tissue per 2 mL digestion solution. Thereafter the digested fat was serially filtered through a final filter size of 100 μm. The filtrate was centrifuged at 1,200 rpm for 5 min and the resulting pellet was suspended in growth medium (DMEM, 10% fetal bovine serum, and 1% antibiotic-antimycotic). Cell suspensions were distributed into T150 tissue-culture flasks. The growth medium was changed every 2–3 days and cells were passaged upon reaching 80–90% confluency. Upon reaching passage 3, tri-lineage differentiation capacity of the cells was confirmed via monolayer culture in (1) osteogenic medium (DMEM, 10% v/v FBS, 1% v/v antibiotic-antimycotic, 0.1 µM dexamethasone, 10 mM β-glycerophosphate, and 50 µg/mL ascorbic acid) and (2) adipogenic medium (DMEM, 10% v/v FBS, 1% v/v antibiotic-antimycotic, 1 µg/mL ITS, 1 µM dexamethasone, and 0.5 mM 3-isobutyl-1-methylxanthine (IBMX)), or pellet culture in (3) chondrogenic medium (DMEM, 1% v/v antibiotic-antimycotic, 10 µg/mL insulin-transferrin-selenium (ITS; Invitrogen, Carlsbad, CA), 0.1 µM dexamethasone, 40 µg/mL proline, 50 µg/mL ascorbic acid, and 10 ng/mL recombinant human transforming growth factor-β3); On day 21, the cultures were stained with Alizarin Red, Oil Red O, or Safranin O/Fast Green using standard histological staining protocols to assess chondrogenesis, osteogenesis, and adipogenesis, respectively [15].

### GelMA preparation

15 g of gelatin (Sigma-Aldrich) was dissolved in 500 mL H_2_O at 37 °C, and 15 mL of methacrylic anhydride (Sigma-Aldrich) was added dropwise. The mixture was incubated with shaking at 150 rpm for 24 h, then dialyzed against water for four days, and methacrylated gelatin was lyophilized for storage. The photoinitiator, lithium phenyl-2,4,6-trimethylbenzoylphosphinate (LAP), was prepared as described by Fairbanks et al. [27]. To fabricate hydrogels, methacrylated gelatin was dissolved in Hank’s Balanced Salt Solution (HBSS) with final concentrations of 15%. The pH was adjusted to 7.4 with 10 N NaOH and 1% antibiotic-antimycotic and 0.15% w/v LAP were added. The stiffness of GelMA scaffolds increases with exposure time, reaching a plateau of compressive modulus of approximately 20 kPa after 4 min of VL exposure, as shown in early work from our group [11]. The tunable stiffness of the utilized GelMA scaffolds has been shown through several studies to support chondrogenic differentiation of expanded MSCs in vitro and in vivo.

### PRP preparation

Thawed ASCs and reconstituted GelMA hydrogels were prepared on the morning of surgery. Following the onset of anesthesia, 9 mL of peripheral blood was drawn from the ear vein of each rabbit. A 1 mL aliquot was collected into EDTA tubes to obtain the platelet concentration and total white blood cell concentration in whole blood. The remaining 8 mL of whole blood was collected into tubes containing 1 mL sodium citrate and immediately centrifuged for 10 min at 200 x *g*. The plasma layer was aspirated, leaving behind the buffy coat and red blood cell layers. The plasma was centrifuged again for 10 min at 1000 x *g*. The supernatant was carefully removed with a remaining plasma volume of 1500 µL and an undisturbed pellet. Using the remaining 1500 µL plasma, the pellet was resuspended, with the resulting solution considered to be leukocyte-depleted platelet-rich plasma (PRP). A 1 mL aliquot of PRP was collected into EDTA tubes to determine the concentrations of platelets and white blood cell concentration and differential.

### Osteochondral defect creation and implantation

**Fig. 1:**
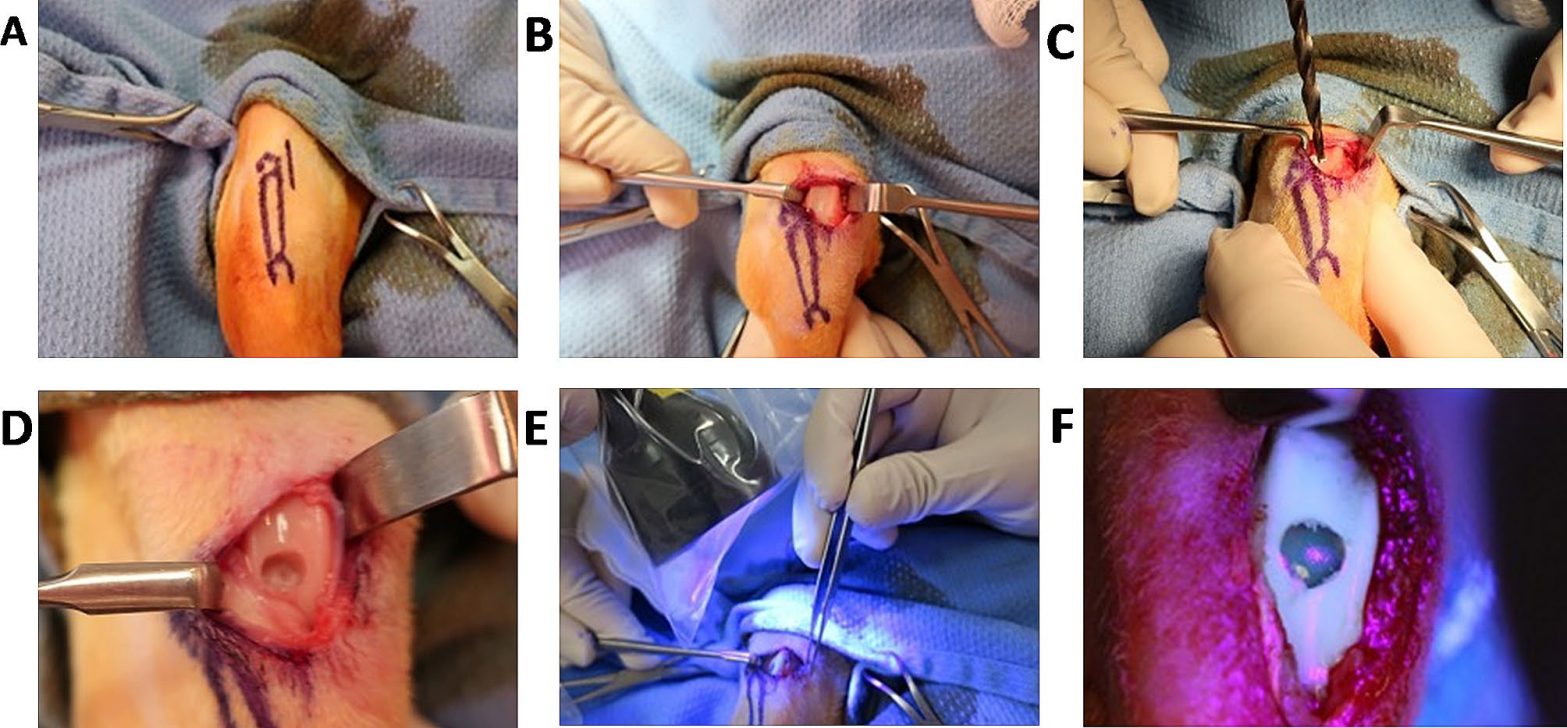
Osteochondral defect creation and hydrogel implantation. (**A**) A medial parapatellar incision was used to access the joint space, (**B**) the patella was reflected laterally to expose the articular surface, (**C, D**) a cylindrical osteochondral defect was created in the patellofemoral groove, (**E, F**) and the liquid hydrogel (+/- ASC and +/- PRP) was polymerized in situ by photo-illumination (395 nm)

All mature New Zealand white rabbit complied with the United States National Research Council’s Guide for the Care and Use of Laboratory Animals and the Committee on Ethics of Medical Research of the University of Pittsburgh Institutional Animal Care and Use Committee (Approval number: Protocol 1,716,357). Thirty rabbits were divided into six experimental groups: (1) Sham - joint capsule was cut open and closed; (2) Defect - osteochondral defect was left untreated; (3) GelMA - defect was filled with 10% methacrylated gelatin (GelMA); (4) GelMA + ASC - defect was filled with ASC suspension at 20 × 10^6^ cells/mL in 10% GelMA; (5) GelMA + PRP - defect was filled with autologous PRP mixed with 15% GelMA at 1:2 volume ratio; and (6) GelMA + ASC + PRP - defect was filled with cell suspension at 20 × 10^6^ cells/mL and PRP in GelMA. Each group had five rabbits and each rabbit underwent bilateral operations (*n* = 10 knees per group). Before the surgery, rabbits were anesthetized with the mixture of ketamine (30 mg/kg) and xylazine (2 mg/kg) by intramuscular injection for the induction of anesthesia, then conducted the intubation attained with 1.5% isoflurane during the procedure. Briefly, the knee joints were opened via a medial parapatellar incision (Fig. 1A). The patella was reflected laterally to expose the patellofemoral articular surface of the joint (Fig. 1B). A critical-sized defect of 5 mm in diameter and 5 mm in depth was created on the trochlear groove (Fig. 1C). When appropriate, 100 µL of hydrogel (± PRP ± ASCs) was used to fill the osteochondral defect. The hydrogel was then subjected to photoillumination (395 nm) for 5 min to induce gelation. The joint capsule and skin were independently closed and the rabbits were recovered from anesthesia and returned to their cages.

### Macroscopic assessment

All rabbits were euthanized by an intraperitoneal injection for pentobarbital overdose and sacrificed 12 weeks postoperatively. The femoral patella groove was dissected, exposed, and photographed. Gross appearance was evaluated according to the ICRS macroscopic assessment score for cartilage repair [28, 29] (Supplementary TableS1).

### Mechanical indentation testing

After photographing the repair site, the distal femur was dissected free of all surrounding soft tissues. The distal femur shaft was cut approximately 5 cm proximal to the joint line then mounted to an aluminum stage using aluminum screws. The apparatus was submerged in a phosphate-buffered saline bath at room temperature and allowed to equilibrate. Strain-controlled indentation testing was performed using a mechanical testing instrument (Bose ElectroForce 3230 Series II) driving an electromechanical indentation probe. The distal end of the rod consists of a protruding flat-ended cylindrical indenter (radius *R* = 525 μm). The flat end of the indenter was positioned orthogonal to the articular surface of the trochlea and the applied compressive load was measured using a 9.8 N load cell. After applying a small preload of 0.1 N (115 kPa), the indenter was lowered at a strain rate of 25 μm/s to a depth of 50 μm (loading phase). The indenter was held at peak displacement for 13 s to allow for stress relaxation (holding phase), and then withdrawn at 25 μm/s (unloading phase). Figure 6B shows a schematic of the indentation procedure on a graph of displacement vs. time as well as a typical load vs. displacement curve and linear fit. Each test was performed on the central part of the defect.

### Indentation analysis

For a rigid flat-ended cylindrical indenter, the commonly applied Hertz theory of linear contact mechanics predicts a linear load-displacement relationship during the loading phase. Due to the geometrical constraints of our experiment, namely the large ratio α between the indenter radius *R* and cartilage thickness *h*, we chose to employ a modification of Hertz’ classic linear elastic theory [30]. While application of Hertz theory treats cartilage as an infinite elastic half space, the theory developed by Hayes et al. is suited for thin cartilage samples and larger indenters with *α* > 1. Hayes theory models cartilage as an infinite elastic layer of thickness *h* in which a non-uniaxial stress field arises due to compression against an underlying rigid half space representing subchondral bone [30]. This model leads to the following expression for the indentation elastic modulus:

$$E=\frac{P(1-{\nu }^{2})}{2R{x}_{0}\varvec{\kappa }(\varvec{\alpha },\varvec{\nu })}$$

where ***κ(α,ν)*** is a compensatory function accounting for layered geometry that depends on the *R*/*h* ratio *α* and Poisson’s ratio *ν*. The ***κ*** function, described by Hayes et al., is solved via a numerical integration kernel with a step size of 0.01. As Hayes and colleagues show, ***κ*** values are greater than 1 for any real value of *α* and for Poisson’s ratios in the range of 0-0.5. Thus, the incorporation of this correction factor decreases the apparent elastic modulus measured via indentation.

Poroelastic materials such as cartilage undergo stress-relaxation during static strain-controlled compression. After allowing stress relaxation to bring a sample to equilibrium an equilibrium elastic modulus *E*_*eq*_ can be calculated from the equilibrium strain and applied load [31]. In this regime, the compressed material is assumed to be linearly elastic, allowing the Hayes equation to be applied [30–32]. To determine equilibrium elastic moduli, samples were indented according to the procedure described above. Load vs. displacement data were collected for all samples and smoothed using a Savitzky-Golay filter. The equilibrium elastic modulus *E*_*eq*_ was calculated using the Hayes equation (above) with *P* representing the equilibrium load during the “hold phase” and *x*_*0*_ representing the applied displacement at equilibrium. A Poisson’s ratio of ν = 0.4 was assumed for all samples.

The equilibrium elastic modulus describes material behavior at equilibrium and upon instantaneous step loading, but values of *E*_*eq*_ can be appreciably lower than other moduli measured during loading [32]. The tangent elastic modulus *E*_*tan*_ describes a stress-strain relationship at any specific point of the loading cycle [32–34]. Tangent elastic moduli may be calculated using the Hayes equation applying assumptions of linear elasticity [32]. Here, the linear loading profiles we observed indicated that the linear elasticity assumption holds experimentally. To determine tangent elastic moduli, load vs. displacement data were smoothed as above and a linear fit was applied to the upper 35% of the “loading phase” curve using linear least-squares regression. A small number of curves consisting of abnormal noise or in which or a linear fit was unable to be obtained (R^2^ < 0.80) were discarded. The slope of the linear regression was extrapolated to calculate *P*/*x*_*0*,_ and *E*_*tan*_ was subsequently calculated using the Hayes equation. A Poisson’s ratio of ν = 0.4 was assumed for all samples, as above. Figure 6B displays how tangent and equilibrium elastic moduli were measured via a linear fit on the upper portion of the loading curve.

### Micro-CT

Micro-computed tomography (micro-CT) scanning (Inveon system from Siemens, 80 kV exposure voltage, and 500 µA current in 2250 ms) was performed to evaluate subchondral bone regeneration in the neotissue of samples harvested. The specimens were fixed in 10% buffered neutralized formalin and prepared for micro-CT and histological analyses. For three-dimensional visualization and analysis of images of new bone formation, the image data was reconstructed using OsiriX MD software (Osirix MD imaging software, Pixmeo, Geneva, Switzerland), rendering a 3D representation of the regenerated bone. Based on the CT data, a cylindrical region of interest (ROI) was analyzed that corresponded to the original defect location. For comparison between groups, the extent of bone regeneration within the defect was calculated as a bone volume/total volume (BV/TV) ratio using a validated threshold [35, 36].

### Histological and immunohistochemical assessment

All samples were fixed in 10% paraformaldehyde, dehydrated using a graded ethanol series, embedded in paraffin, and sectioned at a thickness of 6 μm following a standard histological procedure. Sections were stained with Safranin O/Fast Green (Millipore Sigma, Burlington, MA). To perform immunohistochemistry, deparaffinized and rehydrated sections were incubated with primary antibodies against human collagen type I, collagen type II, or collagen type X (Abcam, Cambridge, MA) at 4 °C overnight, followed by incubation with appropriate secondary antibodies. Immunostaining was carried out using the Vectastain ABC kit and NovaRED peroxidase substrate kit (Vector Labs, Burlingame, CA, USA). Sections from the repair site were examined by two independent reviewers using the modified ICRS II histological scoring system consisting of 11 parameters [37, 38] (Supplementary Table S2). This system is an integrated evaluation of tissue and cell morphology with emphasis on restoration of normal cartilage and subchondral bone plate architecture as well as the integration of the graft with the surrounding host tissue.

### Statistical analysis

All data were expressed as mean ± standard deviation, and statistical analyses were performed using either two-way independent analysis of variance (ANOVA) or two-way independent multivariate analysis of variance (MANOVA), followed by Tukey’s HSD post hoc testing. A threshold of *p* < 0.05 constituted statistical significance.

### Arrive guidelines 2.0

All aspects of this animal research have been reported in line with the ARRIVE guidelines 2.0.

## Results

### Experimental design and composition of PRP

**Fig. 2:**
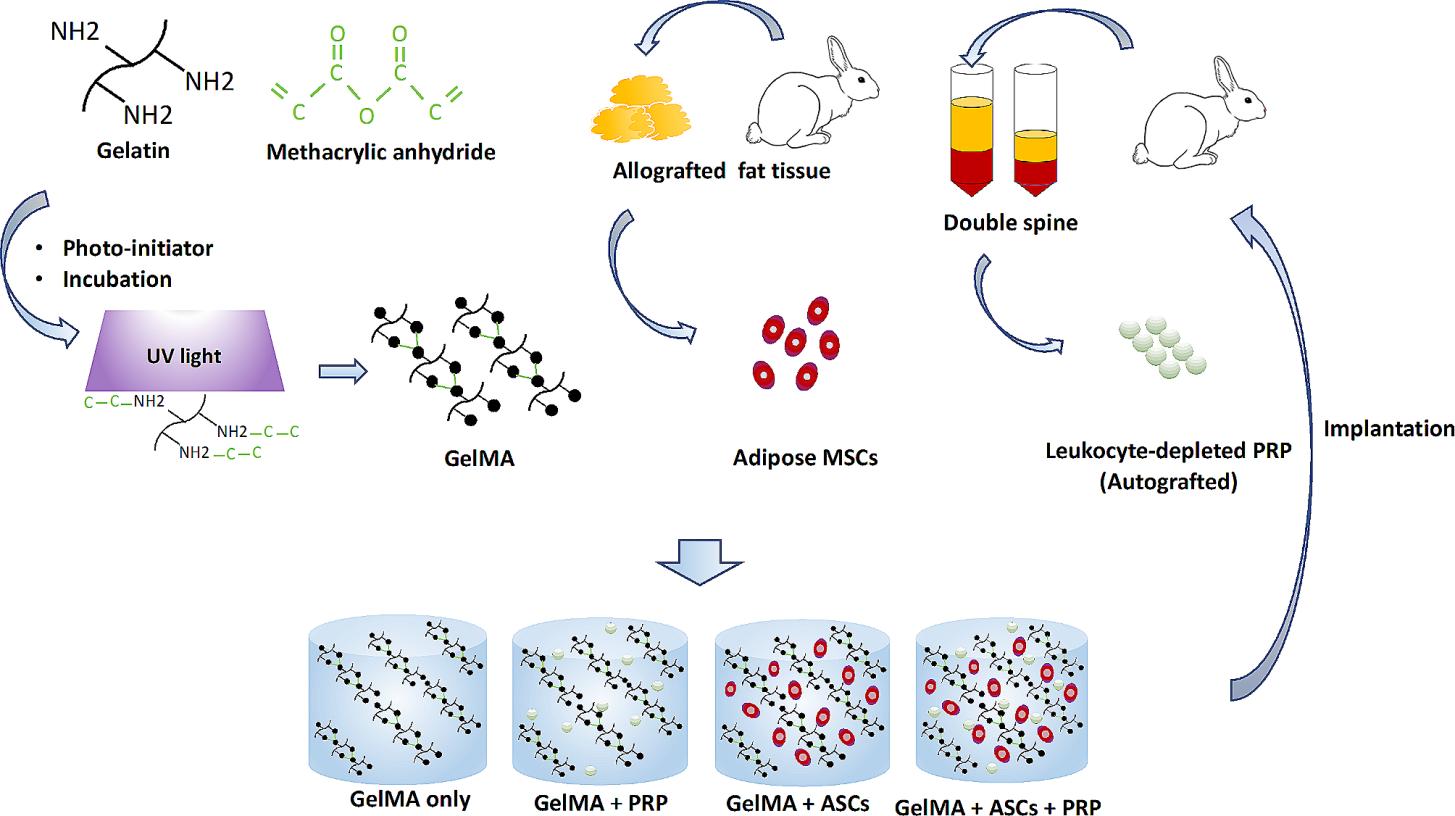
Schematic representation of the photo-crosslinking process of the hydrogel and preparation of the ASCs from fat tissue and PRP from venous blood

**Fig. 3:**
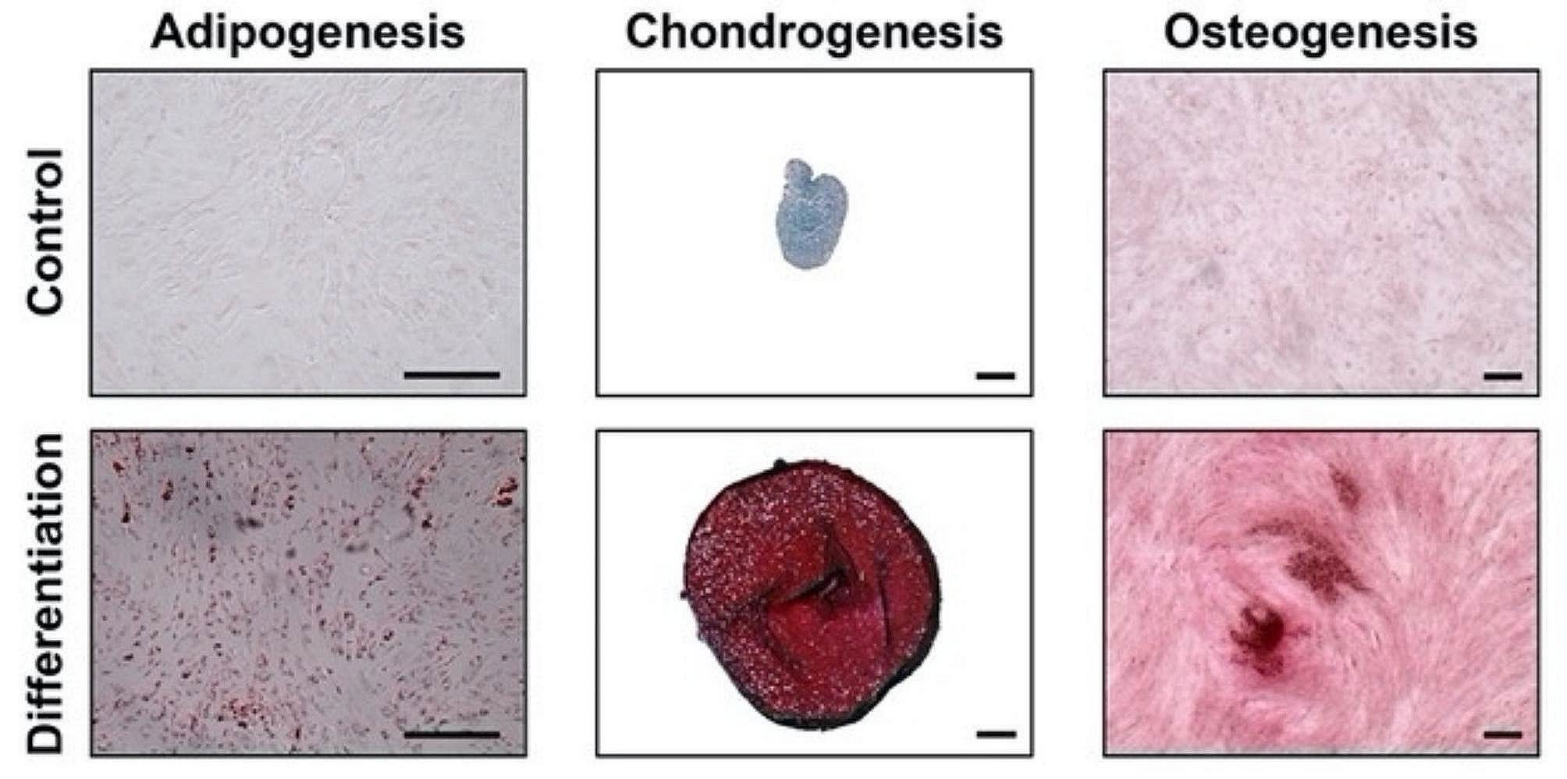
ASCs demonstrated trilineage differentiation capacity, including adipogenesis (monolayer stained with Oil Red O), chondrogenesis (pellet stained with Safranin O/Fast Green), and osteogenesis (monolayer stained with Alizarin Red) from left to right. Scale bar = 200 μm

To investigate the effectiveness of a combination of ASCs and leukocyte-depleted PRP, first, we extracted the subcutaneous adipose tissue from the interscapular adipose of six rabbits (Fig. 2). The mean weight of the donor rabbits was 3.7 ± 0.4 kg, while the mean weight of extracted adipose tissue was 31.7 ± 16.6 g. After enzymatic digestion of connective tissue, followed by filtration and centrifugation, an average of 70 × 10^6^ total cells could be collected, which including numerous cell types comprising the stromal vascular fraction, including ASCs. This heterogeneous cell population was then expanded in growth medium on tissue culture plastic to selectively enrich for ASCs. ASCs demonstrated the capacity to undergo trilineage differentiation (Fig. 3). Cells were cryopreserved until needed.

**Fig. 4:**
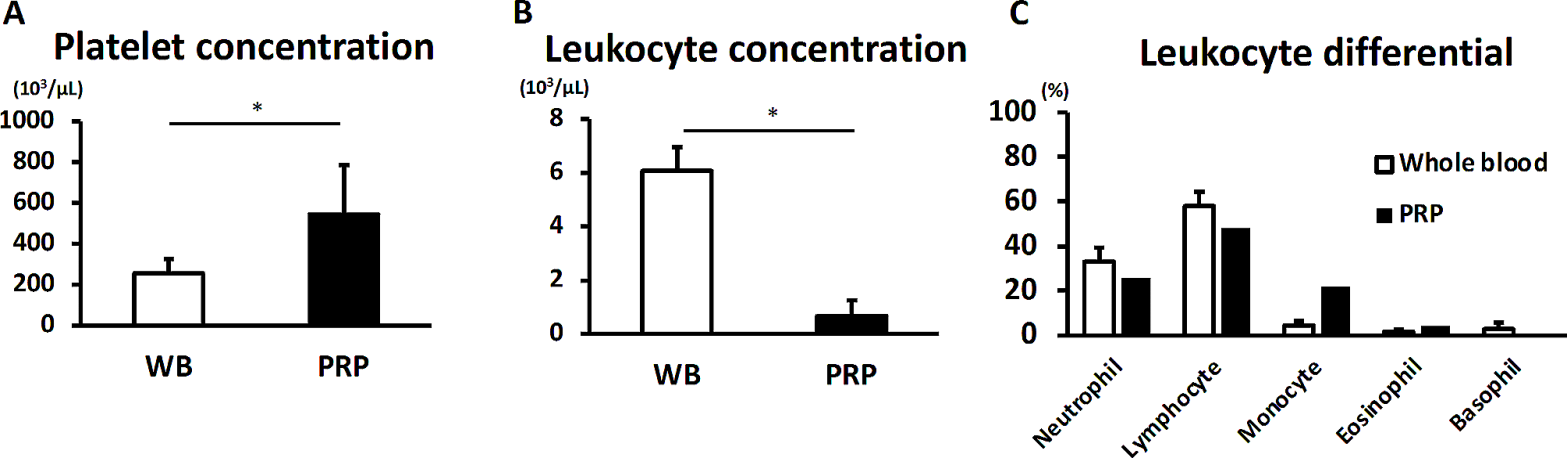
Composition of PRP, including (**A**) platelet concentration, (**B**) leukocyte concentration, and (**C**) leukocyte differential. *, significantly higher compared to platelet number and leukocyte whole blood (*p* < 0.05)

Preoperatively, 9 mL of peripheral blood was drawn from each New Zealand white rabbit. The concentration of platelets in PRP at 544.6 ± 242.3 × 10^3^ µL was significantly higher than that at 255.6 ± 70.4 × 10^3^ µL in whole blood (*p* = 0.009) (Fig. 4A). The mean leukocyte concentration in PRP at 0.664 ± 0.581 µL was significantly lower than that at 6.08 ± 0.879 µL in whole blood (*p* < 0.0001) (Fig. 4B). The leukocyte differential of whole blood was as follows: neutrophils: 32.8 ± 6.4%, lymphocytes, 58.0 ± 6.3%, monocytes: 4.6 ± 1.8%, eosinophils: 1.8 ± 0.8%, basophils: 2.8 ± 2.8%. On the other hand, owing to the much smaller number of leukocytes in PRP, the leukocyte differential could not be detected with the exception of one sample (R693 shown) in Fig. 4C. This result demonstrated that leukocyte-reduced PRP and allogeneic ASCs could be implanted into GelMA in this study.

### Macroscopic evaluation

**Fig. 5:**
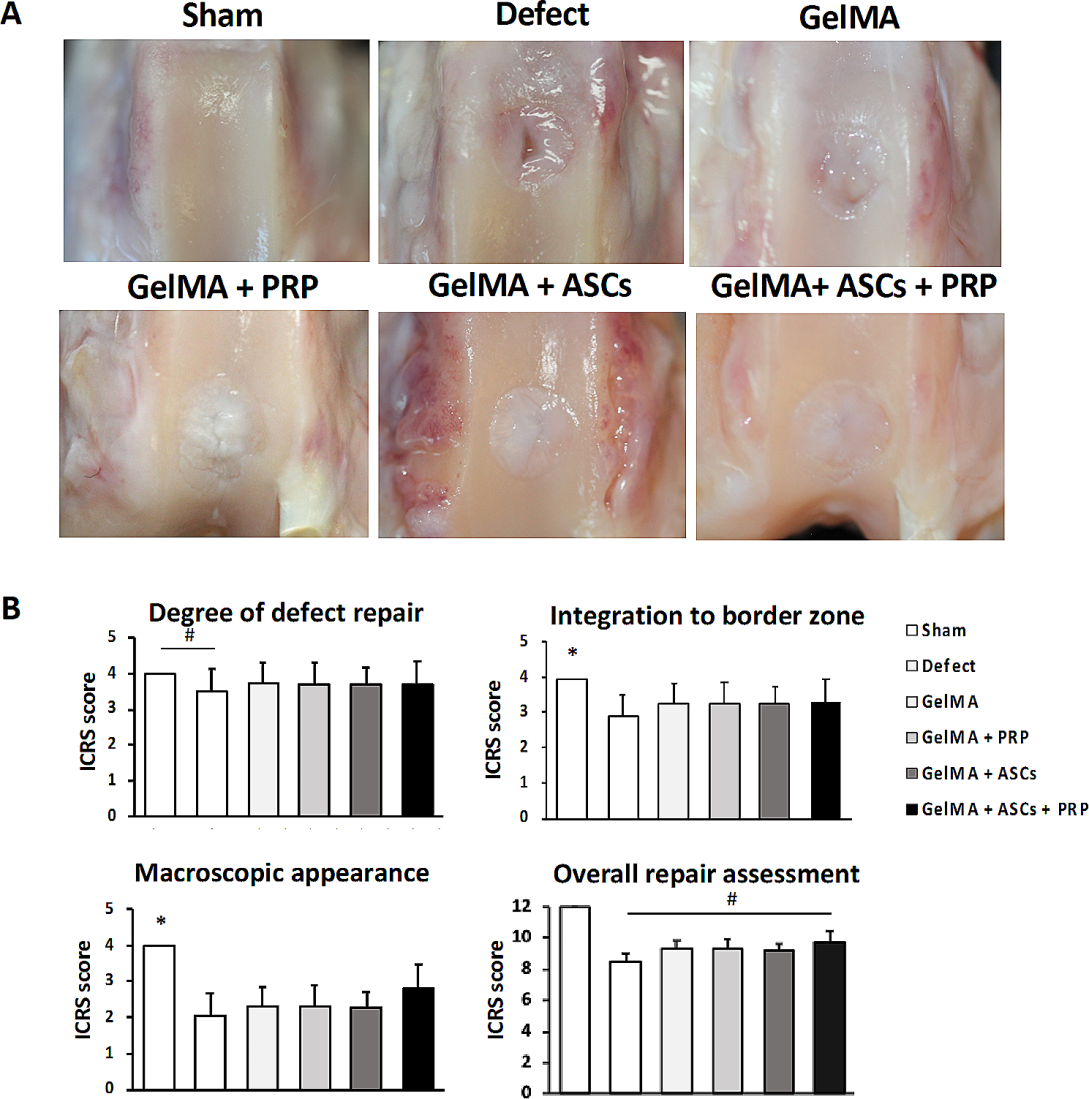
ICRS macroscopical score. (**A**) Macroscopic observations of the full-thickness cartilage defect sites treated GelMA + ASCs + PRP appeared glossy white and suggested fully filled defects. (**B**) For the overall repair assessment, GelMA + PRP + ASCs group resulted in significantly higher scores than the Defect group. #, significantly higher than other groups (*p* < 0.05). *, significantly higher compared to all other groups (*p* < 0.05)

At 12 weeks after surgery, the neotissue in the defect group appeared to be slightly integrated with adjacent cartilage, but inconsistently filled with soft tissue and showed evidence of fissures on the surface at the transplant sites (Fig. 5A). The macroscopic observation of the full-thickness cartilage defect sites in the GelMA + PRP + ASC group revealed glossy white surfaces and fully filled defects as compared with the defect group. (Fig. 5A). The results of our ICRS scoring system for gross macroscopic evaluation was quantitatively analyzed (Supplementary Table S1). For all subcategories in the scoring system, there were no significant difference between three treatment groups and controls. However, for the overall repair assessment, only GelMA + PRP + ASCs group (9.77 ± 1.57) resulted in significantly higher scores than the Defect group (8.45 ± 1.27) (*p* = 0.002) (Fig. 5B).

### Mechanical indentation testing

**Fig. 6:**
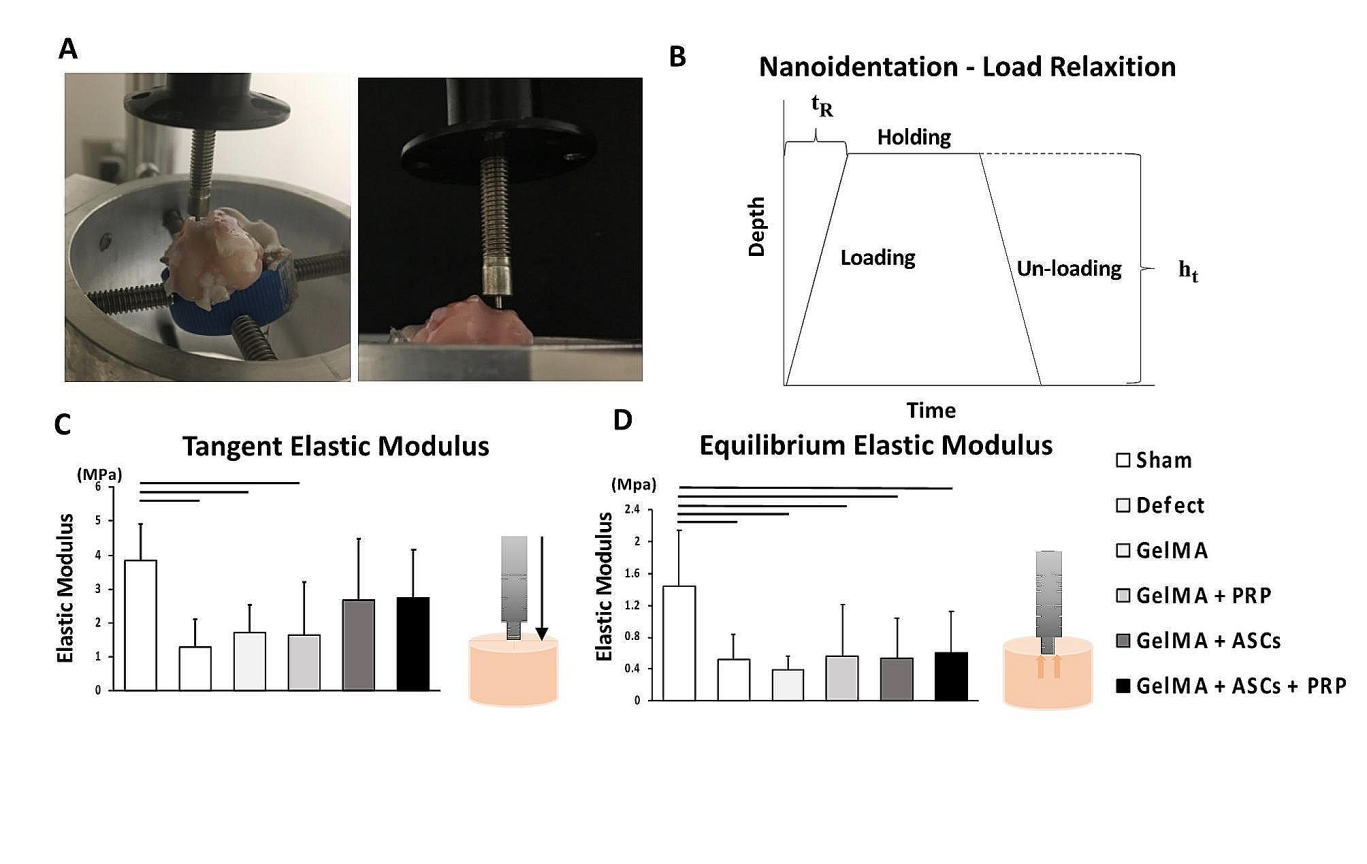
Mechanical properties of repair tissue in osteochondral defects filled with GelMA hydrogels augmented with ASCs, PRP, or ASCs with PRP. (**A**) Schematic of the indentation apparatus and setup. (**B**) Schematic of the indentation procedure showing indenter displacement vs. time as well as a typical load vs. displacement curve and linear fit. The indenter was first lowered at a strain rate of 25 μm/s to a depth of 50 μm (loading phase), then held at peak displacement for 13 s to allow for stress relaxation (holding phase), and finally withdrawn at 25 μm/s (unloading phase). Each test was performed on the central part of the defect. (**C**) Tangent elastic modulus of neo-tissue calculated during the loading phase. (**D**) Equilibrium elastic modulus of neo-tissue calculated during the holding phase. Black bars indicate significantly higher moduli compare to other groups (*p* < 0.05)

After preloading, the indenter was lowered at a strain rate of 25 μm/s to a depth of 50 μm (loading phase). The indenter was held at peak displacement for 13 s to allow for stress relaxation (holding phase) (Fig. 6A, B). The elastic modulus measured during the loading phase, known as the tangent modulus, is a mechanical property that reflects the ability of neotissue to bear stress during strain-controlled dynamic loading. The tangent elastic moduli of the defect groups (1.28 ± 0.84 MPa), GelMA group (1.70 ± 0.82 MPa) and PRP group (1.65 ± 1.56 MPa) were significantly lower than that of the Sham group (3.85 ± 1.04 MPa) (*p* = 0.001, *p* = 0.0066, *p* = 0.0098, respectively). On the other hand, the GelMA + ASCs group and the GelMA + ASCs + PRP group (2.67 ± 1.79 and 2.75 ± 1.41 MPa, respectively) did not exhibit significantly lower tangent elastic moduli than the Sham group (*p* = 0.346, *p* = 0.40, respectively) (Fig. 6C). These results suggested that incorporation of ASCs assists in restoring the ability of the neotissue to bear stresses similarly to native tissue. The elastic modulus measured during the hold phase, known as the equilibrium elastic modulus, represents the ability of neotissue to bear a static load after the matrix has undergone stress relaxation to an equilibrium state. Equilibrium elastic moduli of the Sham group (1.36 ± 0.72 MPa) were significantly higher than the Defect group (0.50 ± 0.35 MPa), GelMA group (0.39 ± 0.17 MPa), GelMA + PRP group (0.54 ± 0.50 MPa), GelMA + ASCs group (0.56 ± 0.65 MPa) and GelMA + ASCs + PRP group (0.61 ± 0.53 MPa) (Fig. 6D).

### Histological and immunohistochemical assessment

**Fig. 7:**
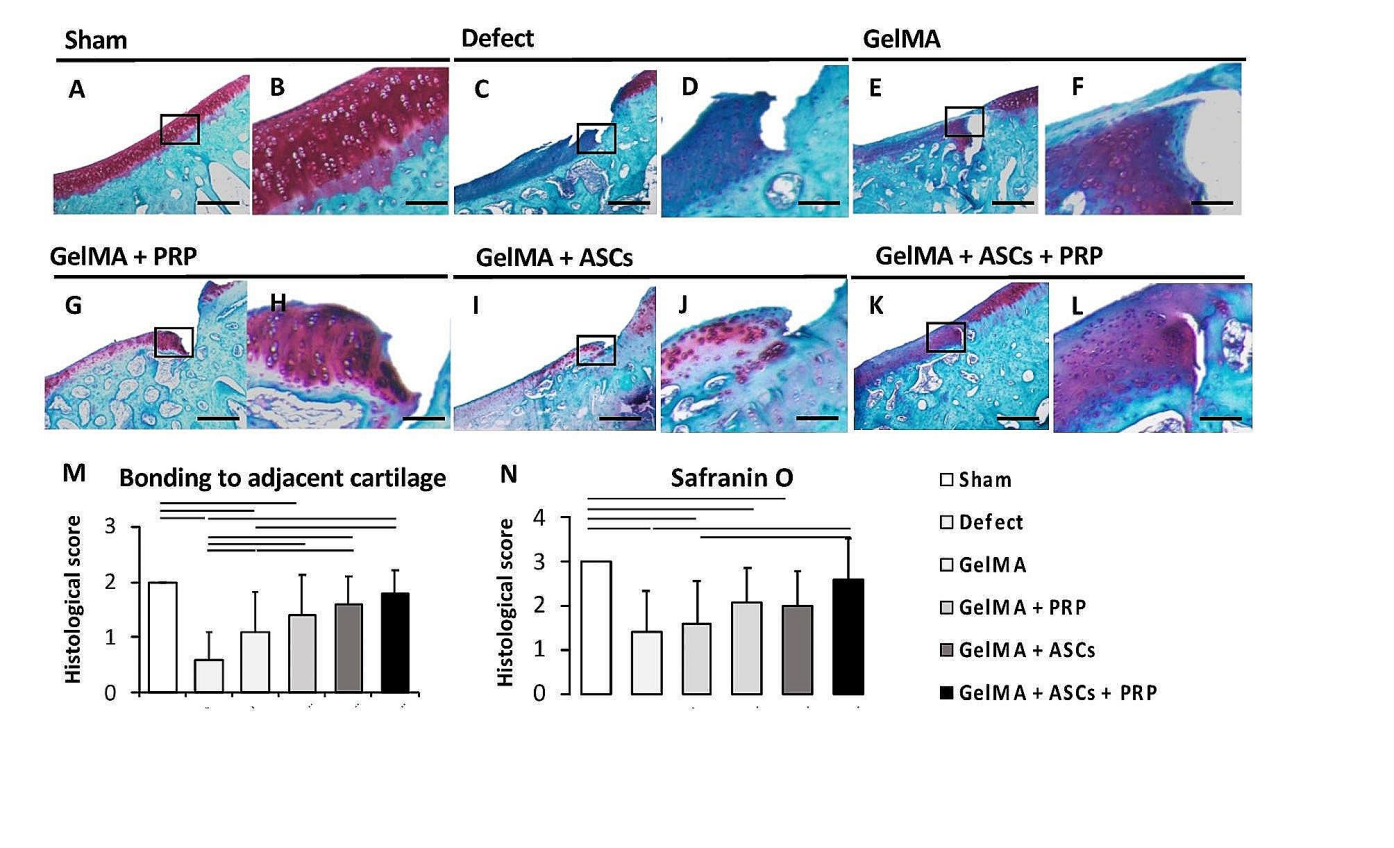
Safranin O staining and modified ICRS II histological scoring. (**A, B**) Sham group, (**C, D**) Defect, (**E, F**) GelMA, (**G, H**) GelMA + PRP, (**I, J**), GelMA + ASCs, (**K, L**) GelMA + ASCs + PRP. For a given group, the left panel is low magnification (scale bar = 100 μm) and the right panel is a higher magnification (scale bar = 10 μm) at the junction of the neotissue and native osteochondral surface. (**M** and **N**) histological scores according to Niederauer et al. [38] (Supplementary Table S2). Black bar, significantly higher compared to other groups (*p* < 0.05)

The GelMA + ASCs + PRP group stained more strongly for proteoglycan content (red stain, Fig. 7K, L) than the defect and GelMA groups (Fig. 7C-F). On the basis of histological scores according to Niederauer et al. [38], safranin O staining in GelMA + ASCs + PRP (2.6 ± 0.94) groups was significantly higher than the defect (1.4 ± 0.94) and GelMA (1.6 ± 0.94) groups (*p* = 0.0009, *p* = 0.0017, respectively) (Fig. 7N). In the GelMA + PRP and GelMA + ASCs groups, smooth and newly formed matrix with proteoglycan existed at the articular surface, however fissures were still frequently observed at the integration of the neotissue and native articular surface (Fig. 7G-J). On the other hand, the integration of neotissue with native tissues in the GelMA + ASCs + PRP group was often seamless and smooth (Fig. 7K, L). Other scores were shown in Supplementary Table S3.

**Fig. 8:**
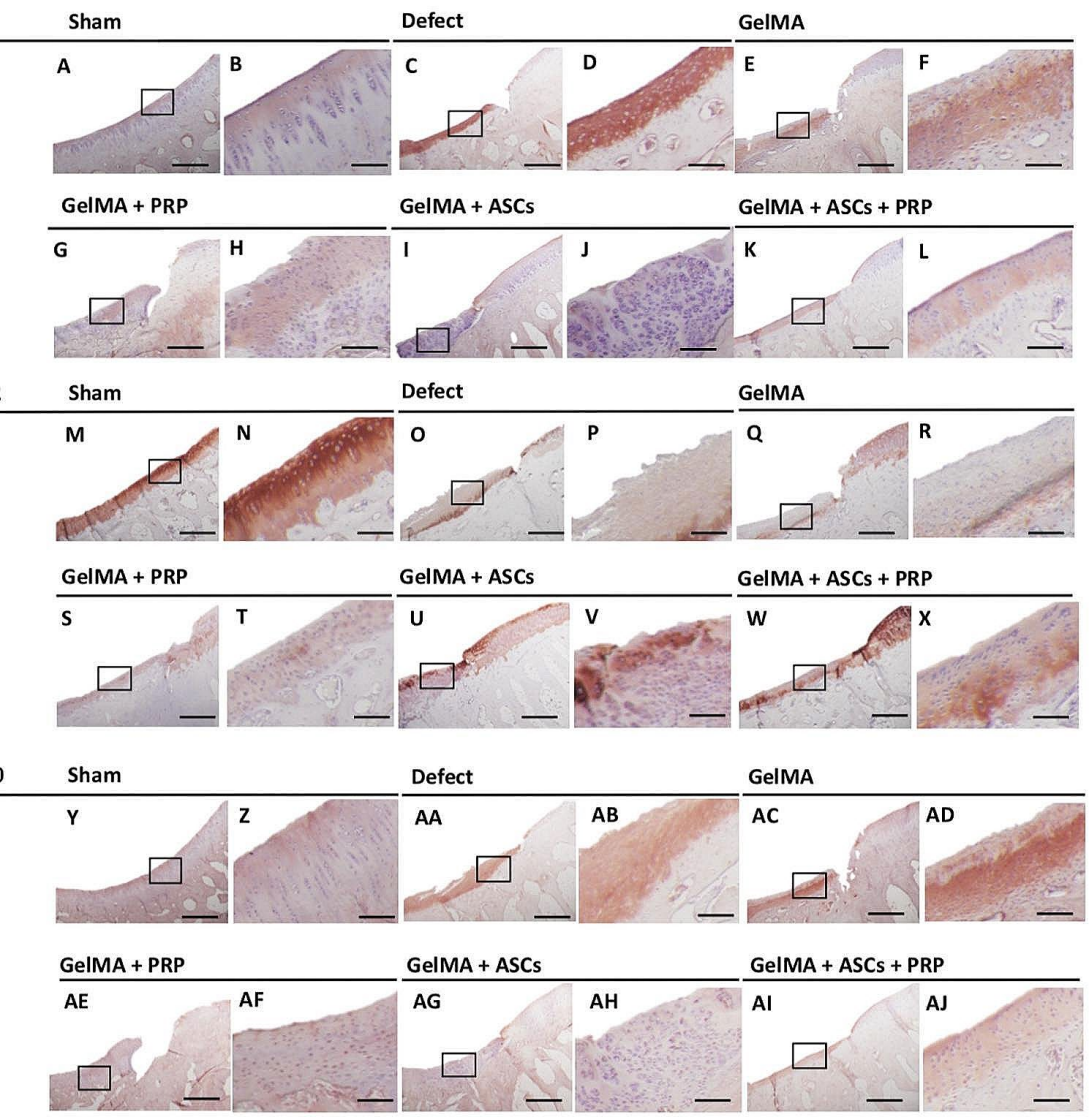
Immunohistochemical staining for collagen type I, collagen type II, and collagen type X. Representative sections showing staining for collagen I (**A** to **L**), collagen II (**M** to **X**), and collagen X (**Y** to **AJ**). For a given group, the left panel is low magnification (scale bar = 100 μm) and the right panel is a higher magnification (scale bar = 10 μm) of the area designated by the black rectangle

Immunohistochemical staining showed that strong staining for collagen type I and collagen type X in the GelMA groups (Fig. 8E, F **and AC, AD**). On the other hand, the content of collagen type I and collagen type X decreased within the neo-cartilage at the transplantation site in the PRP group, ASCs group and ASCs + PRP group, with the ASCs group showing the strongest effect. Additionally, collagen type II content increased as compared to the Defect groups and GelMA groups; the ASCs group and ASCs + PRP group presented especially strong staining for collagen type II, similarly to the Sham group as a positive control (Fig. 8X). Surprisingly, the PRP group and PRP + ASCs groups tended to exhibit higher collagen type I and collagen type X staining compared to the ASCs group, suggesting a potential PRP-mediated hypertrophic effect.

### Analysis of regenerated subchondral bone by microCT

**Fig. 9:**
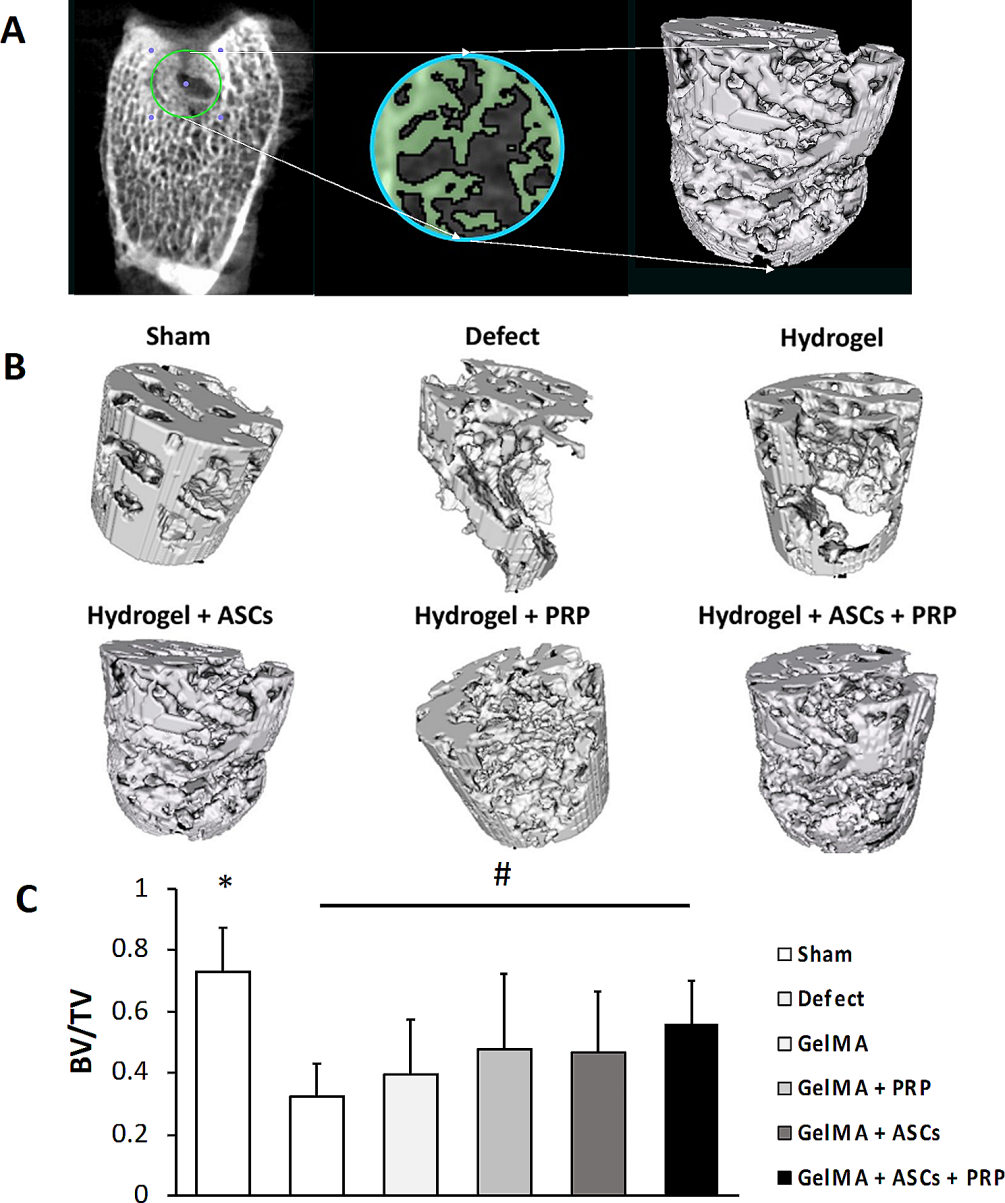
Subchondral bone regeneration analyzed by microCT. (**A**) Schematic demonstrating subchondral bone architecture in a cylindrical region of interest (ROI) corresponding to the original osteochondral defect, as analyzed by microCT. Green color represents regenerated bone and black color represents other tissues in the original defect (3-D model *ex situ* reconstructed by 2-D slice). (**B** and **C**) Comparison of bone regeneration using the ratio of BV/TV. *, significantly higher compared to all experimental groups (*p* < 0.05); ^#^, significantly higher compared to Defect group (*p* < 0.05)

Mineralized bone formation was assessed using microCT scanning. BV/TV in the GelMA + ASCs + PRP group was significantly higher than the defect group *(p* = 0.0126). On the other hand, there was no significant difference between the other treatment groups and the defect group (Fig. 9), indicating that the combination ASCs + PRP enhanced subchondral bone regeneration. All experimental groups had a lower BV/TV compared to Sham controls.

## Discussion

This study found improvement in osteochondral healing mediated by an in situ photocrosslinked hydrogel containing leukocyte-reduced PRP or allogeneic ASCs, with a synergistic effect of combining PRP and ASCs, as assessed by gross appearance, biomechanical properties, histological and immunohistochemical characteristics, and subchondral bone volume. These findings are consistent with previous studies. Notably, Hsu et al. [24] reported that intra-articular injection of both ASCs and platelet-rich fibrin releasate, compared to either alone, improved the gross and histological appearance of healing osteochondral defects in a rabbit model. Likewise, Spakova et al. [25] found improved gross and histological characteristics of healing osteochondral defects in a rabbit model when treated with locally adherent ADSCs and subsequent PRP injections, as compared to microfracture with PRP injections or no treatment. While these results were promising, biomechanical properties, immunohistochemical characteristics, and subchondral bone quality were not assessed. This study is the first to examine the individual and combined effects of PRP and ASCs encapsulated in a photocrosslinkable hydrogel as single-stage treatment for osteochondral defects.

Hydrogels have been used widely for osteochondral tissue engineering, given their ability to deliver high concentrations of homogeneously distributed cells while being amenable for in situ gelation within irregularly shaped defects [39–42]. We chose to use the photocrosslinked methacrylated gelatin hydrogel (GelMA) as a bioscaffold in this study. The advantages of GelMA were highlighted in in vitro experiments by Lin et al. [11], demonstrating that visible light (VL)-based photocrosslinking of the GelMA hydrogel permitted cell encapsulation that led to desirable properties for articular cartilage repair, including cytocompatibility, chondroconductivity, and tunable mechanical properties. In addition, GelMA hydrogels can be rapidly photocrosslinked by VL, an easily adaptable technique for arthroscopic procedures. The promotion of chondrogenesis on MSC-seeded GelMA was previously demonstrated in vitro by Rothrauff et al. [10], who showed upregulated gene expression of chondrogenic markers *ACAN*, *COL2*, and *SOX9*.

In addition to GelMA, other photocrosslinked hydrogel products have been investigated. For example, Pascual-Garrido et al. manufactured a photopolymerizable cartilage mimetic hydrogel modified by chondroitin sulfate and arginyl-glycyl-aspartic acid to enhance cell adhesion and provide chondrogenic cues [43]. Qi et al. designed a sericin methacryloyl (SerMA)-based UV crosslinking hydrogel, which was adhesive to chondrocytes and promoted the proliferation of attached chondrocytes even under a nutrition-deficient condition [44]. In vivo implantation of chondrocyte loaded SerMA hydrogels promoted cartilage formation. Absent direct comparison of differing hydrogels, identification of the optimal biomaterial for osteochondral tissue regeneration remains to be elucidated. Given our laboratory’s extensive experience with GelMA, we used this established biomaterial to explore the additive effects of PRP and/or ASCs for osteochondral regeneration.

PRP, with its myriad bioactive factors that participate in the innate wound healing process, has been investigated as an orthobiologic for osteochondral regeneration [45]. While intraarticular injection of PRP has been recently shown to clinically improve pain and knee function in early osteoarthritic knees [46], likely mediated by the anti-inflammatory and immunomodulatory effects of PRP, its efficacy in promoting neotissue formation in cartilage or osteochondral lesions remains uncertain and has been largely explored only in in vitro and animal models. Notably, while some studies have found PRP to promote chondrogenic differentiation of MSCs in vitro [13], others have found a neutral [16, 47] or inhibitory effect on chondrogenesis [15]. Similarly, treatment of osteochondral defects by PRP alone has yielded equivocal results [13, 48], as this study found GelMA + PRP tended to improve osteochondral healing but seldom reaching statistically significant differences compared to controls.

Rather, the combination of ASCs + PRP produced superior effects, suggesting that the ASCs improved healing either by contributing to neotissue formation and/or secreting paracrine factors that further facilitated endogenous repair beyond the bioactive mediators found in PRP. While the mechanism underlying the added benefit of including ASCs was not investigated, MSCs are principally known to improve healing through paracrine signaling, with few studies convincingly demonstrating neotissue formation by transplanted exogenous cells. Despite the additive benefit of including both ASCs and PRP in the photocrosslinked hydrogels, the regenerated osteochondral tissue did not perfectly recapitulate the structural properties of the native articular surface. While ASCs were utilized herein because they are easier to isolate and are present at a higher density per tissue volume than BMSCs, alternative MSC sources for osteochondral repair, such as BMSC [13] and cartilage-derived chondrogenic progenitor cells [49] have been described and may promote further improvements in osteochondral repair, and are subject of ongoing studies.

Although articular cartilage is known to be a poroelastic material [50, 51], a variety of models have been applied to elucidate the mechanical properties of cartilage in experimental conditions similar to our study [32]. For a rigid flat-ended cylindrical indenter, the commonly applied Hertz theory of linear contact mechanics predicts a linear load-displacement relationship during the loading phase [52]. Our indentation of repair tissue at defect sites demonstrated linear loading profiles and could be suitably fitted using linear elastic theory. Based on these findings, we calculated tangent elastic moduli using the last 35% of the loading phase curve. We additionally sought to capture the behavior of repair tissue after stress relaxation to an equilibrium state, utilizing poroelastic theory to calculate equilibrium elastic moduli after the completion of stress relaxation during the hold phase. Tangent elastic moduli are usually reported to be higher than equilibrium elastic moduli [32], as they represent the dynamic response of cartilage to increasing strain before the material is able to relax. These values are also specific to the rate of loading (25 μm/s in this study). By contrast, equilibrium elastic moduli represent the way cartilage responds to a prolonged static load, such as during standing. To gain a more clinically relevant picture of cartilage mechanics, both dynamically measured tangent moduli and static equilibrium moduli should be reported when possible. Here, our results showed no significant differences in tangent elastic moduli between the Sham group and the GelMA + ASCs and GelMA + ASCs + PRP groups, suggesting that incorporation of ASCs assists in restoring the ability of the neotissue to bear stresses similarly to native tissue. However, all repair groups showed lower equilibrium elastic moduli than the Sham group, suggesting that repair matrices are less able to bear stress once relaxation has occurred and water has evacuated the matrix. Modulation of the mechanical microenvironment of the healing osteochondral tissue, such as the use of biomaterials with different mechanical properties and/or controlling in vivo loading through the use of bracing or time-dependent rehabilitation protocols, may further improve the restoration of native osteochondral biomechanical properties [53, 54]. These constructs composed of alternative biomaterials or loading parameters could first be explored using in vitro bioreactors so as to better optimize design parameters before their in vivo application.

This study was not without limitations. While the rabbit model used herein has been commonly employed in similar studies, it does not fully recapitulate the mechanical forces and healing microenvironment of the human, which may be better accomplished through the use of a larger animal model. Furthermore, only a single timepoint was explored, limiting our understanding of the effect of the studied constructs on osteochondral regeneration over time. A single ASC density (20 × 10^6^ cells/mL) was used; different concentrations may impact osteochondral regeneration, although the studied concentration was previously shown to produce abundant cartilage matrix [55]. The platelet concentration in PRP, while enriched over whole blood, was not controlled per se, and differing platelet concentrations may affect PRP bioactivity. Finally, this study did not explore the mechanisms by which ASCs and PRP exert a beneficial effect on osteochondral regeneration. Further understanding of the pathogenesis of osteochondral injury and the limited innate healing capacity of the native articular surface will in turn inform therapeutic strategies, including biologics such as PRP and ASCs. Mechanistic studies are ongoing.

## Conclusion

Regeneration of a focal osteochondral defect in a rabbit model was improved by a single-stage treatment of a photocrosslinked hydrogel containing allogenic ASCs and autologous PRP, with the combination of ASCs and PRP producing superior benefit than either alone. At the single studied timepoint of 12 weeks after surgery, no treatment group fully restored the gross appearance, biomechanical properties, histological and immunohistochemical characteristics, and bone volume of the neotissue to that of native osteochondral tissues.

### Electronic supplementary material

Below is the link to the electronic supplementary material.

Supplementary Material 1

## Data Availability

The datasets generated and analyzed during the current study are available from the corresponding author on reasonable request (IT, RST).

## Abbreviations

ASCs: adipose-derived mesenchymal stromal cells
PRP: platelet-rich plasma
GelMA: methacrylated gelatin hydrogel
ACI: autologous chondrocyte implantation
TGF-β: transforming growth factor-β
VEGF: vascular endothelial growth factor
PDGF: platelet-derived growth factor
IGF: insulin growth factor-1
b-FGF: basic fibroblastic growth factor
DMEM: Dulbecco’s Modified Eagle’s Medium
ITS: insulin-transferrin-selenium
FBS: Fetal bovine serum
