# Supplementary material for: Enhanced osteochondral repair by leukocyte-depleted platelet-rich plasma in combination with adipose-derived mesenchymal stromal cells encapsulated in a three-dimensional photocrosslinked injectable hydrogel in a rabbit model
Source: Stem Cell Res Ther. 2024 Jun 3;15:159. doi: 10.1186/s13287-024-03750-z (PMC11149275; doi:10.1186/s13287-024-03750-z)
Supplement: Supplementary file 1 — Supplementary Material 1 [file 13287_2024_3750_MOESM1_ESM.docx]

**Supplementary Materials**

**Supplementary Table S1:** International cartilage repair society (ICRS) macroscopic scoring system

**Supplementary Table S2:** Histological scores according to Niederauer et al [37]

**Supplementary Table S3:** The results of histologically evaluated for each group using the histological scoring system
